# Fatal Overdose due to Confusion of an Transdermal Fentanyl Delivery System

**DOI:** 10.1155/2013/154143

**Published:** 2013-04-02

**Authors:** Ingo Voigt

**Affiliations:** Elisabeth Krankenhaus Essen, Klinik für Kardiologie und Angiologie, Klara Kopp Weg 1, 45138 Essen, Germany

## Abstract

*Background*. The use of transdermal fentanyl systems has increased over recent years, especially in patients with chronic pain. Large misuse potential and fatal outcomes have been described. *Case Presentation*. A 58-year-old patient presenting with clinical signs of opioid poisoning (hypoventilation, bradycardia, hypotension, and miosis) was admitted to our ICU. The first body check revealed a 75 mcg per hour fentanyl patch at the patient's right scapula. Some months ago, patient's aunt died after suffering from an oncological disease. During breaking up of her household, the patches were saved by the patient. Not knowing the risk of this drug, he mistook it as a heat plaster. *Investigations*. Laboratory test showed an impaired renal function and metabolic acidosis. Urine drug test was negative at admittance and 12 h later. CCT scan presented a global hypoxic brain disease. *Treatment and Outcome*. The patient was discharged 30 days after admittance in a hemodynamic stable condition but a vegetative state and transferred to a rehabilitation center. *Learning Points*. With the ongoing increase in fentanyl patch prescriptions for therapeutic reasons, it is likely that misuse cases will become more relevant. Conventional urine drug screening tests are not able to exclude the diagnosis fentanyl intoxication. History taking should include family member's drug prescriptions.

## 1. Background

The use of transdermal fentanyl delivery systems has increased over recent years especially in patients with chronic pain who are already treated with high doses of morphine or it is derivate. Fentanyl patches, which provide steady-state fentanyl concentrations for 72 hours, are an attractive alternative treatment compared to multiple daily oral medications especially in geriatric and cancer patients. However, a large misuse potential with fatal outcomes has been described [1–3]. The minority of incidents occur in places with controlled and documented patch administrations such as hospitals or retirement centers. On the contrary, no control exits in a residential setting. 

## 2. Case Presentation

A 58-year-old mechanically ventilated patient was admitted to our ICU. His wife reported that when she arrived home after work, she found him unresponsive lying in bed. Unable to feel any pulse at all, she called the emergency department and started basic cardiopulmonary resuscitation. Upon arrival of the emergency doctor, the patient presented with hypoventilation, bradycardia (45 bpm), and severe hypotension (60/30 mmHg). First documented oxygen saturation was 60% and GCS was 3. After unproblematic oral intubation without any need of further narcotics, the patient was transferred to our ICU. 

## 3. Investigations 

Biochemistry results demonstrated an impaired renal function (GFR 22 mL/min/1.7 m²), metabolic acidosis (pH 7.21) with an BE of −7 mmol/L, and a high potassium level of 6.2 mmol/L. Sodium, chloride, lactate, and albumin were in normal range. Further blood tests including a full blood count, liver function, and coagulation test showed no abnormalities. At admission and after 12 hours, an urine drug-screening test (TOX/See; Bio-Rad, USA) was negative for amphetamine, barbiturate, benzodiazepine, cocaine, marihuana, methadone, methamphetamine, MDMA, opiate, oxycodone, phencyclidine, and tricyclic antidepressant. 12-channel ECG and echocardiography ruled out acute myocardial infarction, pericardial tamponade, pulmonary embolism, and aortic dissection. No diagnostic signs of a septic shock were found. The first neurologic investigation confirmed a GCS-Score of 3. Additionally, a miosis was found. To rule out intracranial hemorrhage, a head CT was performed immediately. This scan showed a slight diffuse cerebral edema but no intracranial hemorrhage or early signs of ischemic stroke. 

During the transport back from the CT-scanner, the ICU doctor noticed a translucent patch at the patient's right scapula. After removing the patch, it was identified as a 75 mcg per hour fentanyl transdermal-delivery system (Durogesic, JANSSEN-CILAG GmbH). 

Summing up the symptoms of hypoventilation, bradycardia, hypotension, unconsciousness, and miosis, we diagnosed opioid intoxication by a fentanyl patch. Because medical history did not reveal a reason for prescription of such a highly potent opioid, the wife of the patient was interviewed again. She reported that her husband suffered from moderate pain of the right shoulder after a bike trip with friends some days ago. However, she did not know about her husband using a fentanyl patch. Further inquiry revealed that some months ago the aunt of the patient died after suffering from an oncological disease. During the breaking up of her household, the fentanyl patches were saved by the patient. By thinking that these patches may relief pain like an over-the-counter heat plaster, he probably underestimated the potential danger of this drug. The night before ICU admission, the patient must have attached the transdermal fentanyl patch to his hurting back and went to bed. When his wife woke up early for work, she did not recognize the fatal overdose because it was normal that her husband is still asleep when she leaves home. 

## 4. Treatment and Outcome

The primary therapy included respiratory support with mechanical ventilation and the administration of noradrenaline to reach an MAP of >65 mmHg. Because the patient was already intubated, we did not administer naloxone as antidote concerning the short duration of action. In combination with a positive fluid balance renal parameters went back to normal. No other organ failure occurred. Unfortunately after the discontinuation of analgosedation by tearing of the fentanyl patch, the patient presented general seizures needing an anticonvulsive therapy with valproic acid. A follow-up head CT 72 h later still showed a global hypoxic cerebral edema but no intracranial bleeding or signs of ischemic stroke. The neurological consultation confirmed a hypoxic brain damage. In preparation for a prolonged rehabilitation, a percutaneous dilatational tracheotomy was performed at day 7 to ease ventilator weaning, and a PEG-tube was implanted for gastric feeding. 

The patient was discharged 30 days after admittance and transferred to a rehabilitation center in a hemodynamic stable condition, though in a persistent vegetative state. 

## 5. Discussion

Although the product's patient information leaflet states that these patches are intended only for patients who are already treated with opioid agents of comparable dosage, misuse is reported in some cases. In an autopsy study, 25 cases of deadly fentanyl intoxications and the postmortem tissue distribution have been described [1]. All of them were former drug addicts or patients suffering from severe oncological disease who misused it for various reasons. On the other hand, several cases of accidental and even deadly intoxications of children have been reported, for example, caused by incorrect disposal of used patches in open trashcans.

In this case the accidental intoxication happened to an academic adult who mistook the Durogesic patch as an over-the-counter heat plaster. Although the product's patient information leaflet states that the patch needs narcotic prescription, this information is not present on the wrapping of the patch itself. In this case presentation, the patches were bequeathed by the deceased aunt, and no information of the potential risk of these patches was available. This raises the question who is liable for redemption of unused fentanyl patches if a patient dies? In Germany, the narcotic prescription law provides regulations for rehabilitation centers, retirement homes, or palliative care institutions but no advice is mentioned for at home prescriptions as presented in this case report [4]. 

Second, this case report reveals another not well-known fact that fentanyl use or misuse cannot be diagnosed by a simple urine drug test. Most widely used tests are based on an immunoassay technique [5]. They have a good sensitivity and specificity to diagnose typical recreational drugs like marihuana, cocaine, benzodiazepine, or amphetamine. By using monoclonal antibodies elevated morphine, codeine and heroine level can be detected in humane urine. Fentanyl, alfentanil, sufentanil, remifentanil, and their major metabolites cannot be detected by the monoclonal antibodies used in these urine drug tests. To rule out fentanyl use effectively, a urine drug test based on liquid chromatography and tandem mass spectrometry should be chosen [6].

## 6. Learning Points

With the ongoing increase in fentanyl patch prescriptions for therapeutic reasons, it is likely that misuse cases will become more relevant.

Conventional urine drug screening tests are not able to exclude the diagnosis fentanyl intoxication. A test based on liquid chromatography and tandem mass spectrometry should be used.

History taking should include family member's drug prescriptions.
